# Virtual Practical Examination for Student Nurse Educators in Health Sciences Education during the COVID-19 Pandemic: A Narrative Review

**DOI:** 10.3390/nursrep13010021

**Published:** 2023-02-08

**Authors:** Kholofelo Lorraine Matlhaba

**Affiliations:** Department of Health Studies, University of South Africa, Pretoria 0002, South Africa; matlhkl@unisa.ac.za; Tel.: +27-12-429-2073

**Keywords:** student nurse educators, e-assessment, virtual practical examination, health sciences education

## Abstract

(1) Background: There is a gap in the literature that explores challenges and opportunities relating to virtual or e-assessment health science education with particular relevance to the Health Sciences Education practical examination for student nurse educators. Therefore, this review aimed to address this gap and provide recommendations for enhancing identified opportunities and for overcoming identified challenges.; (2) Methods: The review was conducted across Google Scholar, PubMed/MEDLINE, Science Direct, Directory of Open Access Journals, Complementary Index, SCOPUS, and the Cumulative Index to Nursing and Allied Health Literature (CINAHL) with the intention of identifying opportunities and challenges presented by e-assessment in the HSE practical examination for student nurse educators during the COVID-19 pandemic.; (3) Results: The following aspects are discussed: (1) opportunities, including benefits, for both student nurse educators and facilitators and opportunities for Nursing Education; and (2) challenges, including issues with accessibility and connectivity as well as the attitudes of both students and facilitators.; (4) Conclusions: Despite challenges which included connectivity issues that led to frustration and stress, the unpreparedness and attitudes of students and facilitators, there are some opportunities that have emerged from e-assessment that can be beneficial to both the students and the facilitators, as well as the institutions. These include a reduced administrative burden, improved teaching and learning, and immediate feedback from facilitators to students and from students to facilitators.

## 1. Introduction

Assessment is a crucial element of any effective teaching and learning strategy [1] at all stages of education, particularly in higher education in the Human and Health Sciences. Within teaching and learning in the Health Sciences, physical examination skills are essential to the practice of clinical care, and students traditionally study and practice their physical skills in person in a particular setting, because this involves considerable time spent on hands-on learning [2]. The same notion is shared with Nursing Education, whereby student nurse educators practice their teaching skills and are assessed on those skills.

In March 2020, the World Health Organization (WHO) declared the highly infectious and deadly COVID-19 disease to be a worldwide pandemic. The COVID-19 pandemic, first confirmed in December 2019, is defined as the worldwide spread of a disease caused by a new coronavirus labelled SARS-CoV-2 [3]. In this review, pandemic refers to the worldwide spread of that disease which forced a sudden change and transition from physical assessment strategies to online or virtual strategies. The majority of higher education institutions including health profession educators (HPEs) across the world had to transition from physical teaching and learning strategies to online strategies for emergency remote learning [4]. Continued pandemic restrictions imposed on face-to-face learning resulted in a decision to permanently transition a graduate nursing education advanced assessment course from a hybrid one to online learning [5,6,7,8,9]. With online learning, learning takes place partially or entirely over the Internet, making it an ideal course delivery model for adult learners wishing to develop new skills and competencies [10]. It is noticeable that online learning has become one of the most popular educational alternatives to meet the demands of today’s global knowledge economy [11].

### 1.1. Background

Online assessment is a system that involves assessments through the web or intranet [12]. Supporting the above-mentioned definition, [13] define online assessment as an e-system that involves assessment of students in an online context. The use of technology in assessment first began in the 1920s, and e-assessment enhances the measurement of learner outcomes, making it possible for them to obtain immediate and direct feedback [7]. With e-assessment, online or virtual assessment is witnessing significant changes to compliment the e-learning strategies established as a result of the pandemic. In this narrative review, the terms online assessment, virtual assessment and e-assessment are used interchangeably. Online assessment is used to assess applied knowledge and skills that can be assessed online [14].

Among their other roles and competencies, nurse educators are expected to execute professional teaching of knowledge and skills to facilitate teaching and learning, enable learner development, and supports learners’ continuous life-long learning [15]. In this regard, the student nurse educators are assessed on their skills in preparation of their journey to becoming competent nurse educators. It has been suggested that there is a need for formal preparation for nurse educators who foster the ongoing development of nurses in clinical practice; therefore, nurse educators, regardless of the setting in which they work or are preparing to work, must receive formal instruction about online teaching [16].

### 1.2. Online or Virtual Practical Examination

There are several virtual or online practical examinations assessed in the literature including virtual streaming and screen activity recordings. These online practical examinations are conducted through the form of live streaming, video-conferencing style platforms that provide the opportunity to have several participants at a time. These platforms are mainly laptop and/or mobile phone app based, where an individual speaking directly to the camera on a mobile phone has the facility to invite several people to chat on-screen simultaneously.

Based on the literature, video conferencing seems to be the method utilised for medical and nursing students’ virtual examinations. Several web-based video-conferencing platforms have emerged over the last decade that deliver audio, video, and screen-sharing experiences across various devices, enabling users to host webinars, virtual meetings, video demonstrations, video-conferences, and online training [17]. Video conferencing, as another method of virtual practical examination, offers assessors the potential for distance assessment of student skills. Online practical examinations such as webinars enhance students’ knowledge and confidence, and have increasingly been adopted for continuing medical education [18]. It is suggested that due to their wide accessibility, webinars are a way of conferencing that can facilitate learning while ensuring high quality at low cost. Furthermore, webinars enable teachers to share information with students anywhere and at any time using different Internet-capable devices [19]. However, even though the use of video-conferencing has been utilised in the past by some HSE institutions, common barriers reported with this type of virtual assessment include instability of connection and lack of on-site technology and instructional design support. This narrative intends to provide an understanding of the synthesised opportunities and challenges presented by online practical examinations that emerged during the COVID-19 pandemic.

### 1.3. Health Sciences Education (HSE) Practical Examination for Student Nurse Educators

HSE is a specialised area that prepares students for a variety of careers related to medicine, dentistry and nursing. Assessment in HSE has become extremely critical, and learner assessment regimes need to have the capacity to accurately evaluate competences that include attitude, skills and the knowledge acquired during the training of healthcare professionals [20]. In South Africa, as in other countries, professional nurses who are intending to specialise in nursing education must undergo additional training at an accredited university in order to be registered as nurse educators with the South African Nursing Council (SANC) [21]. HSE is part of an undergraduate Bachelor of Arts in Nursing Science degree (BA CUR) qualification that consists of 10 modules including a one-year practical module with 12 credits [22]. The module aims “to enable students to practise the didactical skills of HSE in a simulated teaching environment” [22]. The training may take one to three years, depending on whether the intention is to acquire an advanced diploma only or a degree qualification that allows the graduates to practise as Nurse Educators post completion. The programme is offered via satellite transmission with limited face-to-face interaction with educators from an Open Distance e-Learning (ODeL) institution. In their study, [10] explored the effects of the length of online nurse educator courses and stated that online distance education is an effective strategy to increase nurses’ access to nursing degrees and build program capacity.

Traditionally, the HSE practical examination involved face-to-face interactions conducted either in an actual or a simulated classroom, depending on the university requirements, and which take place through physical interaction between the student nurse educator, the students and the facilitator. It is common knowledge that the nursing education practical is primarily delivered through a traditional means of instruction, including face-to-face classroom instruction and clinical experiences in various practice settings [23]. Nursing education has been in alignment with the constructivist view, which believes that learning takes place via interaction with others [24], as cited by [25]. According to Summers (2017) [26], “teaching requires a skill set of its own”. It is for that reason that learning how to teach and facilitate knowledge acquisition in nursing students requires preparation and additional formal education for nurse educators to be competent in their teaching role [26]. This preparation was previously conducted in face-to-face settings. However, as the need to maintain a safe physical distance during the pandemic rapidly increased, the online provision of health professions education accelerated technology adoption in academic settings [27]. The same method was adopted for the HSE practical examination.

The aim of this paper is to present a comprehensive narrative review synthesising the opportunities and challenges presented by e-assessment in the HSE practical examination for student nurse educators during the pandemic. Furthermore, we aim to make recommendations as to how to overcome identified challenges and to promote identified opportunities.

### 1.4. Problem Statement

A nurse educator plays an important role in promoting student learning and professional development, as well as in offering high-quality nursing education [28]. Keating, Berland, Capone et al. (2021) [29] suggest that the capacity of effective nurse educators is a significant constraint when addressing the global shortage of nurses. According to the WHO [30], the preparedness and expectations of nursing graduates will continue to evolve rapidly as a result of social and demographic changes, increasingly complex healthcare needs and chronic conditions, threats of emerging infectious diseases, and environmental and climate related illnesses. The core competencies of nurse educators include competence in nursing practice, pedagogical competence, communication, collaboration skills, monitoring and evaluating, management, and digital technology [31]. According to SANC, the competencies of a nurse educator are classified into seven domains, with facilitation of learning being the first competence which includes the use of information technologies to skilfully support the teaching-learning process [21]. For registration after qualification, each student nurse educator is required to obtain a minimum number of clinical practicum hours as part of their training that provides opportunities to develop the core competencies that align with course and program outcomes [32].

Prior to the COVID-19 pandemic, it was a SANC requirement that student nurse educators be assessed on theoretical lessons as well as clinical lessons. During the assessment, a real class scenario was simulated with fellow students or at an accredited nursing education institution with student nurses. Students would be assessed by a registered nurse educator on issues that included lesson plan preparation, class facilitation including material used for facilitation and class control to meet the nurse educators’ core competencies as stipulated by WHO [31]. To conform to government-imposed physical distancing regulations for restricted infection transmission [33], the method of assessment was changed. This led to a shift from the face-to-face practical examination to an online practical examination assessment. To overcome pedagogical challenges posed by the COVID-19 pandemic, pre-recorded instructional videos, narrated PowerPoint presentations, and live practical classes with students practising at home are some examples of the digitally enhanced teaching approaches adopted by health science educators to teach practical skills remotely [34]. Naik, Deshpande, Shivananda et al. (2021) [35] reported that in efforts to combat this inevitable crisis, educational sectors began conducting online classes and this sudden changeover in the teaching and learning method raised new challenges and opportunities.

Given the background, it is noteworthy to report that much has been achieved with respect to online teaching and learning in HSE. However, none of the currently available studies have focused specifically on virtual practical examinations for student nurse educators. Therefore, this review aims to synthesise evidence that describes those challenges and opportunities presented by e-assessment in the HSE practical examination for student nurse educators during the COVID-19 pandemic. Furthermore, we aim to make recommendations that might help higher education institutions, particularly those offering nursing education, to enhance the use of e-assessment for practical examinations as an essential assessment tool rather than being one only to be used in such emergency situations as the COVID-19 pandemic.

### 1.5. Definition of Key Concepts

**Human Sciences:** This involves the study and understanding of human beings [36] and nursing as a human science focuses on the humaneness of the person and seeks to provide patient centred care which is directed towards improving the life of a unique individual [37].

**Higher Education**: Higher education is viewed as a vehicle for intellectual development, developing a flexible mind and, regardless of the field of study, helping students acquire knowledge and intellectual skills that can be applied in a variety of different contexts [38]. Higher nursing education is nursing education specifically offered in a university setting, with the aim of preparing nursing graduates with complex knowledge and skills [39].

**Nursing Education** refers to the professional education for the preparation of nurses to enable them to render professional nursing care to people of all ages, in all phases of health and illness, in a variety of settings. According to SANC, Nursing Education is a “specialist field that focuses on education and training students who are undertaking undergraduate and or postgraduate programme in nursing” [21].

**Nurse Educator** refers to a professional with an additional qualification in Nursing Education and is registered as such with the SANC [21]. In this review, the term student nurse educators refers to those professional nurses who are studying to obtain their basic degree qualification in Nursing Education, as explained in the definition above.

### 1.6. Review Purpose

The purpose of this narrative review was to synthesise the current evidence on opportunities and challenges presented by e-assessment in the HSE practical examination for student nurse educators during the COVID-19 pandemic.

### 1.7. Review Question

This narrative review aim to answer the following question:

What are the opportunities and challenges presented by e-assessment in the HSE practical examination for student nurse educators during the COVID-19 pandemic?

## 2. Materials and Methods

A comprehensive literature search was carried out on Google Scholar, PubMed/MEDLINE, Science direct, Directory of Open Access Journals, Complementary Index, SCOPUS, and Cumulative Index to Nursing and Allied Health Literature (CINAHL) with the intention of identifying opportunities and challenges presented by e-assessment in the HSE practical examination for student nurse educators during the COVID-19 pandemic. The search was conducted between August and November 2022.

### 2.1. Data Collection

Narrative reviews, also referred to as literature reviews, are a method used to identify and consolidate that which has been previously published on a specific topic; this consolidation prevents duplication and allows identification of any omissions or gaps for potential new studies [40,41]. This narrative review was conducted following the four steps as suggested [41]. The four proposed steps of narrative review are as follows: (1) a systematic search process and application of inclusion and exclusion criteria; (2) data extraction and synthesis of results; (3) the analysis of key findings by the narrative review; and (4) a quality appraisal procedure that included all studies. For the purpose of this narrative review, this method allowed a thorough search for extant literature, integration and interpretation of findings from varied study types which covered diverse online or virtual practical examinations or assessments in the fields of HSE and the quality appraisal of those studies. 

### 2.2. Inclusion and Exclusion Criteria

The selection criteria included the following: (i) studies and reports written in English that reported on opportunities and challenges of e-assessment or online/virtual assessment in health science education or nursing education; (ii) articles and reports published in peer-reviewed journals; and (iii) published between 2020 and 2022. The search terms used were online practical assessment; virtual practical assessment; e-assessment practical tests; electronic assessment practical; digital practical assessment; virtual practical examination; student nurse educator; student nurse lecturer; student faculty nurse; student nurse teacher.

Articles and reports were excluded if they (i) focused only on e-learning and teaching; (ii) focused on virtual simulation assessment but were published before 2020; and/or (iii) were published in non-peer-reviewed journals. Thesis and dissertations outside health sciences institution repositories were also omitted.

### 2.3. Selection Process

**Figure 1:**
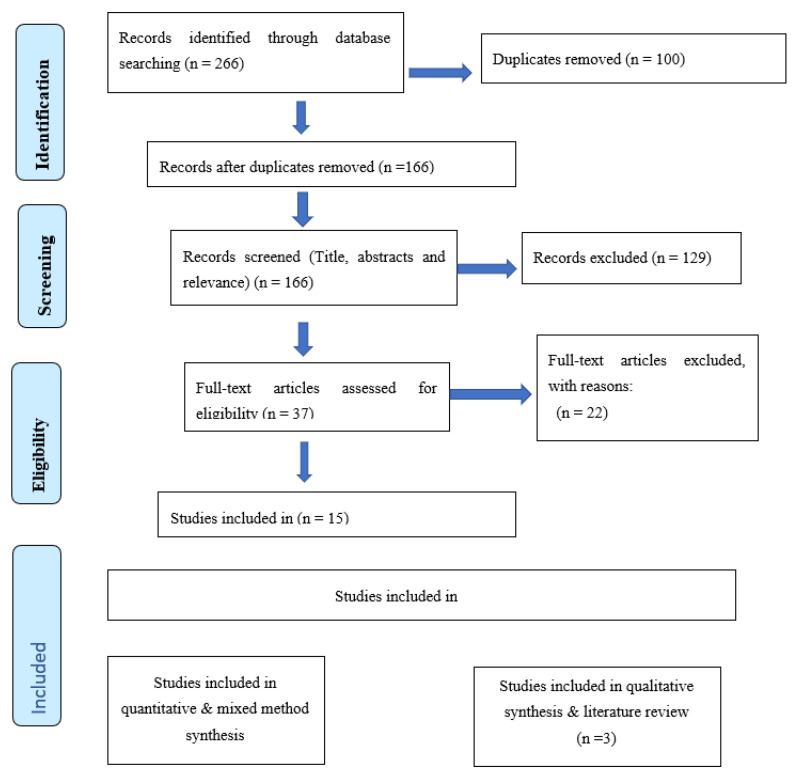
Flow diagram of the narrative review [42].

The Preferred Reporting Items for Systematic Reviews and Meta-Analysis were applied in order to determine the most appropriate articles for review [42]. The search focused on full articles that included the key concepts. The thorough search generated 266 results, including reports and research articles; after duplicates were removed, the remaining 166 were reviewed at title, abstract and relevance level. This review resulted in the further removal of 129 articles. The remaining 37 were closely read to verify the methodology and the population, resulting in the further removal of 22 records. Fifteen articles then remained for critical review. The PRISMA diagram in Figure 1 illustrates the steps followed in selection of included articles. 

### 2.4. Data Extraction, Analysis, Synthesis and Quality/Critical Appraisal

Data were extracted on study details (author/s and country of study), methods (population, sample and sample size, collection and analysis methods), key results and conclusions. Studies were organised and tabulated into classifications by area of specialised fields. The data were analy sed using inductive and descriptive synthesis [43]. The review question was used as the basis for searching the data for relevant expressions, which were then further tabulated. To synthesise the data, the author used tabulation to search the data for similarities and differences and further organise the data into categories named according to the content. The author critically evaluated the final articles using the Joanna Briggs Institute Critical Appraisal tools for qualitative and quantitative studies. The JBI tool with ten items was applied to rate the quality of qualitative studies [44], whereas another JBI tool with eight items was used to evaluate the quantitative (cross-sectional) studies [45]. Finally, 15 studies that met the quality appraisal criteria were retained (n = 15). Table 1 shows the JBI for quantitative and mixed method studies included (n = 12). The included qualitative articles, presented in Table 2, scored yes (n = 2), and the single literature review is presented in Table 3.

### 2.5. Ethical Considerations

The review was conducted ethically throughout the conceptualisation, planning, implementation and dissemination phases. No permission was needed to conduct this review; however, all sources used are duly acknowledged in the text and in the reference list.

## 3. Results

On the basis of the studies included in this review, e-assessment was shown to be controversial among scholars due to the opportunities and challenges presented during the COVID-19 pandemic. Despite all the challenges, which include connectivity issues leading to frustration and stress and the unpreparedness and attitudes of students and facilitators, there are some opportunities that emerged from e-assessment that are believed to be beneficial to both the students and the facilitators, as well as the institution. Opportunities include reduced administrative burden, improved teaching and learning and immediate feedback from facilitators to students and from students to facilitators.

### Details of Empirical Studies

Details of the 15 studies reviewed from the different fields of HSE are indicated in Table 4, below.

## 4. Discussion

The focus of this paper is primarily on the virtual practical examination of student nurse educators in HSE, although related experiences from other graduate nursing education and other multi-disciplinary fields during the COVID-19 pandemic are included where relevant. Assessment is considered an integral part of the learning process. Traditional assessment methods are often based on the student being treated as an isolated individual with limited access to resources and other people [61]. The evolution of technology together with the interruption from the COVID-19 pandemic, new opportunities for assessment are explored to acknowledge the increasingly important role e-assessment is playing in Higher Education. The discussion section will be based on the opportunities or advantages as well as challenges or disadvantages.

### 4.1. Opportunities

Virtual teaching and learning and assessment platforms in HSE, including nursing education, provide innovations and growth opportunities for both students and facilitators. Online practical assessments are innovative in a new reality for most student nurses and teachers and may empower students’ nurses by helping them to remove perceived barriers in face-to-face assessments [58]. Technology assessments such as videos and web-based simulation for advance practice programs in the nursing education institutions were considered due to limited access to healthcare facilities during a period of social distancing [62]. Online practical examination such as telecommunication technology simulation can be an effective strategy to assess clinical skills competencies and provides personalised effective and immediate feedback to students [47,54]. Furthermore, it is suggested that online evaluation had benefits and expected impacts on student and teacher happiness and performance during the COVID-19 pandemic [63], and also improved the teaching and learning process both in managing distance education, increasing class size and staff workload [48,50]. Despite the concerns that included unfamiliarity and limited virtual assessment experience and the insufficient number of information technology technicians that interferes with proper digitalisation [48], the convenience of virtual clinical assessment including the removal of travelling to examination centres provides opportunity to partake at the comfort of both students and the examiners’ homes [59]. As learning to teach and facilitate knowledge acquisition requires preparation and additional formal education to ensure competency in teaching [26], collaboration to ensure effective online assessment practical examination is critical [52,55,64].

### 4.2. Challenges

Virtual practical examination requires sufficient and effective logistics preparation including students’ and facilitators’ training on how to run and partake in the assessment, internet access and proper connections, availability of ICT structural support, as well as infrastructures. Moreover, conducting practical examinations is a challenging aspect during the COVID-19 pandemic; however, virtual-based practical assessment sessions can help teachers to conduct practical examinations effectively [65]. Practical examinations have a role in protecting patients [66] and it is important to continue their delivery via innovative methods of measuring the same knowledge, understanding, and capabilities with amended assessments such using video conferencing platforms to conduct vivas [66].Additionally, problems with internet connection and other technical aspects, the attitudes of teachers, limited interpersonal relations, limited learning of practical skills, health concerns, students’ engagement and distractions during assessment [49,56,67,68] were reported to be worrying factors. The use of virtual practical examination requires abundant preparation on the part of both student nurse educators and the facilitators. Therefore, these issues are undeniably contributory factors to high stress levels experienced by both students as well as the facilitators in HSE during online assessment [12,51]. Furthermore, financial costs and time consumption as the preparation requires more time than the traditional assessment method [69,70] remain part of the challenges. Teachers would require the acquisition of new skills to use digital tools and designing of significant evaluation activities to be used, whereas students are forced to acquire digitals skills that will enable them to use new forms of education [71]. To overcome challenges presented by virtual practical examination during the COVID-19 pandemic is to acknowledge and find ways to deal with those challenges which include collaboration among relevant stakeholders [60,72].

Another challenge presented by virtual practical examination was the question as to whether this method was relevant for some content in HSE. Instances where students could not feel the structure using their hands during virtual practical examination made it difficult for them to identify the structure, its relations and its vascular supply [57,60,73]. It can be argued that despite the positive perceptions, online examinations are not suitable for assessing the physical examination skills [46]. To overcome this challenge, academics in HSE have the responsibility to design and implement strategies that will ensure that the objectives of online practical examination are met which will lead to competent practitioners after completion.

### 4.3. Limitations

There may be some possible limitations in this study. Therefore, the author acknowledges several limitations related to the methods. Narrative reviews are known to be biased in nature [41]. Firstly, although the narrative reviews does not necessarily follow a systematic approach like other reviews [74], the author took steps to attempt to prevent bias by describing the methods followed during including literature search and selection, and discussion of results. However, despite the fact that there is no strict rule on the number of authors required to conduct a narrative review [74] being a single author, she may have been biased during the review process. Secondly, the review only included studies published between 2020 and 2022, which led to limited number of studies that met the set criteria, whereas there were other Pandemics prior to COVID-19. Finally, the majority of the studies used in this review focused on the perceptions and experiences of students and academics regarding virtual practical examination in other health sciences disciplines. This is an indication of a gap in the virtual assessment of practical examinations for student nurse educators. Therefore, the results cannot be generalised, but might provide a framework for future research.

### 4.4. Recommendations

Although educational technologies are increasingly being used in HSE, there is the question as to whether or not it is possible to completely substitute the traditional assessment method [75], particularly for HSE practical examinations. Khoshnevisan (2019) [76] suggests that the technological tools are predominantly far from achieving authentic interaction, and related literature has illustrated that many of these tools do not foster genuine interaction. For these reasons, the level of competence can still be explored. Therefore, because the objectives of the Nursing Education practical module is to prepare a fully competent nurse educator who is able to meet the teaching and learning needs of the students in an increasingly digital, networked world, it is paramount that the effects of virtual practical examinations as an assessment strategy on the competence of nursing educators is identified and explored in further research. Secondly, there is a need for further research into the perceptions and experiences of student nurse educators as well as those of the facilitators regarding online assessment during practical examination in the midst of the COVID-19 pandemic to fully understand the opportunities and challenges presented in HSE.

## 5. Conclusions

Based on the literature reviewed, the researcher concluded that the majority of HSE institutions adopted an online mode of assessment for their students. However, factors such as lack of infrastructure and computer literacy either from the students or from the facilitators can hinder the use of virtual assessment as an essential tool, especially with the HSE practical examination. The uncertainties brought about by the outbreak of the COVID-19 pandemic mandated that HSE facilitators propose durable distance assessment strategies that would minimise physical contact but still maintain the real class presentation skills for student nurse educators to complete the academic year. However, despite all the challenges outlined in this review, the author believes this study provides relevant insights into the opportunities brought by online assessment in the HSE practical examination. By eliminating these challenges, online assessment can be improved, causing it to be of great benefit to the student nurse educators as well as those student nurses who will be in the hands of these educators on completion of their speciality in order to meet the educational needs during and after the pandemic crisis. This is supported by the conclusion that suggested that transformative change in medical education using technology for assessment offers new opportunities for students and that the benefits of the pandemic need to be enhanced in a post-COVID era [53].

### Significance of This Study

The shift from face-to-face practical examination to the virtual mode has gradually been adopted and implemented in HSE and its challenges and opportunities, implications and effects including the competence of graduates need to be thoroughly investigated. Even though this paper was based on the author’s experiences of virtual practical examination, this study highlights the need for innovative ways to conduct and assess practical examination for student nurse educators in HSE during and beyond the Pandemic. The study also suggests that, despite the number of challenges presented by virtual practical examination in HSE, this pedagogical approach can still provide innovation and growth opportunities for both students and facilitators. This means that introduction and implementation of new modern pedagogical approaches such as online practical examination is of great significance and can be used post-pandemic. This study also suggests that since the majority of HSE institutions including nursing education has significantly adopted online teaching and learning, it is important to have future educators who are knowledgeable and comfortable with the use of technology in order to meet the learning needs of the future students. Therefore, virtual practical examination for student nurse educators is a better method to prepare for the task in nursing education.

## Figures and Tables

**Table 1:** Critical appraisal checklist for the included quantitative and mixed method studies.

Study/Criterion	[46] Alkhateeb, Ahmed, Al-Tawil et al. (2022).	[47] Donn, Scott, Binnie et al. (2021)	[48] Elzainy, El Sadik and Al Abdulmonem (2020)	[49] Fatima, Idrees, Jabeen et al. (2021)	[50] Ghaheri, Maghsoudi, Mobarak et al. (2022) Iran	[51] Fogg, Wilson, Trinka et al. (2020)	[52] Kuravi, Gogineni, Bhargav et al. (2021)	[53] Patra and Tekulapally (2021)	[54] Phillips, Munn and George (2020)	[55] Polancich, Steadman, Moneyham et al. (2021)	[56] Przymuszała, Zielińska-Tomczak, Kłos et al. (2022)	[57] Sadeesh, Prabavathy and Ganapathy (2021). India
1. The criteria for inclusion in the sample was clearly defined	U	U	Y	Y	Y	U	Y	Y	Y	Y	Y	Y
2. The study subjects and the setting were described in detail	Y	Y	Y	Y	Y	Y	Y	Y	Y	Y	Y	Y
3. The exposure was measured in a valid and reliable way.	Y	Y	Y	Y	Y	Y	Y	Y	Y	Y	Y	Y
4. Objective, standard criteria were used for measurement of the condition	Y	Y	Y	Y	Y	Y	Y	Y	Y	Y	Y	Y
5. Confounding factors were identified?	Y	Y	Y	Y	Y	Y	Y	Y	Y	Y	Y	Y
6. Strategies to deal with confounding factors were stated	Y	Y	Y	Y	Y	Y	Y	Y	Y	Y	Y	Y
7. The outcomes were measured in a valid and reliable way	Y	Y	Y	Y	Y	Y	Y	Y	Y	Y	Y	Y
8. Appropriate statistical analysis was used	Y	Y	Y	Y	Y	U	Y	Y	Y	Y	Y	Y

Source: JBI Critical Appraisal Checklist for Analytical Cross-Sectional Studies (2017). Key: Yes = Y; No = N; Unclear = U; Not Applicable = N/A.

**Table 2:** Critical appraisal checklist for the included qualitative studies.

Study/Criterion	[58] Roman et al. (2022)	[59] Thampy et al. (2022)
1. Aim and objectives clearly described	Y	Y
2. Research methods appropriate	Y	Y
3. Research design appropriate to address the aim	Y	Y
4. Recruitment of participants adequately described.	Y	Y
5. Data collection addressed.	Y	Y
6. Relationship between researcher and participants has been adequately considered	Y	Y
7. Ethical issues adequately taken into consideration	Y	Y
8. Data analysis sufficiently rigorous	Y	Y
9. Findings clearly described	Y	Y
10. Value of the research is adequately described	Y	Y

Source: JBI Critical Appraisal Checklist for Systematic Reviews and Research Syntheses (2017). Key: Yes = Y; Cannot tell = CN; No = N.

**Table 3:** Critical appraisal checklist for the systematic reviews and research syntheses.

Study/Criterion	[60] Forde and Obrien (2022). Ireland
1. Is the review question clearly and explicitly stated?	Y
2. Were the inclusion criteria appropriate for the review question?	Y
3. Was the search strategy appropriate?	Y
4. Were the sources and resources used to search for studies adequate?	Y
5. Were the criteria for appraising studies appropriate?	Y
6. Was critical appraisal conducted by two or more reviewers independently?	Y
7. Were there methods to minimise errors in data extraction?	Y
8. Were the methods used to combine studies appropriate?	Y
9. Was the likelihood of publication bias assessed?	Y
10. Were recommendations for policy and/or practice supported by the reported data?	Y
11. Were the specific directives for new research appropriate?	Y

Source: JBI Critical Appraisal Checklist for Systematic Reviews and Research Syntheses (2017). Key: Yes = Y; No = N; Unclear=U; Not Applicable = N/A.

**Table 4:** Summary of included articles.

Author/s/Country	Aim	Methods	Participants/Sample Size	Results
1. Alkhateeb, Ahmed, Al-Tawil et al. (2022). Iraq [46]	To share the experience of conducting an online assessment with the academic community and to assess its effectiveness from both examiners’ and students’ perspectives.	A cross-sectional study	Examiners & medical students	The response rates among examiners and students were 69.4% and 88.5%, respectively.
2. Donn, Scott, Binnie et al. (2021) UK [47]	A pilot of a Virtual Objective Structured Clinical Examination (VOSCE) in dental education. A response to COVID-19	Quantitative	Undergraduate dental students	With careful planning, the VOSCE is a useful assessment method in difficult times. Feedback from staff and students was favourable.
3. Elzainy, El Sadik and Al Abdulmonem (2020) Saudi Arabia [48]	Experience of e-learning and online assessment during the COVID-19 pandemic at the College of Medicine, Qassim University	Descriptive cross-sectional study	Undergraduate medical students and staff	The study observed higher student achievements and promising staff perceptions with obvious improvement in their technological skills. These findings support the shift towards future implementation of more online medical courses.
4. Fatima, Idrees, Jabeen et al. (2021) Pakistan [49]	To evaluate online assessment in undergraduate medical education: Challenges and solutions from a LMIC university	Cross-sectional study	Medical students	The students reported that attempting the online exam on VLE with ZOOM support was user friendly. Ninety percent of the class was supportive of the continuing with the online assessments.
5. Ghaheri, Maghsoudi, Mobarak et al. (2022) Iran [50]	Evaluation of Medical Students’ Satisfaction with the Virtual Assessment of Cardiac Physiology Course	Quantitative	Medical students	The students preferred summative assessment questions to be multiple-choice due to the difficulty of the cardiac physiology course. More research should be conducted on this subject with a larger sample size in future studies.
6. Fogg, Wilson, Trinka et al. (2020) USA [51]	To develop evidence-based recommendations for simulation hour equivalence ratios and compile a list of virtual activities and products faculty could use to complete clinical experiences.	Survey	Undergraduate and graduate nursing students	Tailoring learning opportunities such as continuing education courses, open-lab technology sessions, and appropriate reference materials can help to ensure faculty are prepared should the need for online transition be required again in the future.
7. Kuravi, Gogineni, Bhargav et al. (2021) India [52]	Evaluation of experience with virtual conduction of semester practical exams for medical graduates.	A Prospective study	Medical students & Examiners	No problems occurred except a few short-duration (less than 5 min) interruptions due to internet connectivity issues. A total of 125/150 (83.5%) medical students and all examiners (2 internal and 2 external) expressed satisfaction with virtual medical evaluation.
8. Patra and Tekulapally (2021) India [53]	Second-year dental students’ perception of effectiveness of formative assessment in an online learning environment during COVID-19 pandemic	A cross-sectional study	Dental students	Immediate and faceless feedback in the form of a summary of overall performance was preferred by most of the students.
9. Phillips, Munn and George (2020) USA [54]	To evaluate the impact of incorporating telehealth simulation into objective structured clinical examinations (OSCEs) in the family nurse practitioner (FNP) and bachelor of science in nursing (BSN) programs.	Mixed-methods study	Nurse Practitioner students	Students’ telehealth knowledge, skills, and confidence were improved after the telehealth OSCE experience.
10. Polancich, Steadman, Moneyham et al. (2021) US [55]	Unexpected COVID-19 opportunity: applied experience for nurse educator students	Programmatic evaluation, using a 10-item Likert scale evaluation tool	Nurse Educator students	Aggregate mean evaluation scores ranged from 2.7 to 4.3. The nurse educator students attributed an aggregate mean of 4.3 to the possibility of spending additional clinical hours providing oversight to nursing students participating in this process.
11. Przymuszała, Zielińska-Tomczak, Kłos et al. (2022) Poland [56]	Distance learning and assessment during the COVID-19 pandemic—pperspectives of Polish medical and healthcare students	Online questionnaire	Medical Students	Students noticed positive aspects of online learning. However, they also noticed its disadvantages.
12. Sadeesh, Prabavathy and Ganapathy (2021). India [57]	Quantifying students’ experience with virtual assessment.	Quantitative study	Medical students	Completed feedback forms were submitted by 228 students. More than 50% of students favoured online anatomy spotter examinations.
13. Roman, Ruiz-Gonzalez, Rodriguez-Arrastia et al. (2022) Spain [58]	To explore nursing students’ perceptions of the use of a serious game-like model in their final online objective structured clinical examination (OSCE).	Qualitative study	Nursing students	The two main themes were (i) generating emotions and feelings in times of virtuality; and (ii) online assessment: a potential alternative to educational barriers.
14. Thampy, Collins, Baishnab et al. (2022) UK [59]	Virtual clinical assessment in medical education: an investigation of online conference technology	Qualitative study	Medical students	Four themes were identified, namely: optimising assessment design for online delivery, ensuring clinical authenticity, recognising and addressing feelings and apprehensions, and anticipating challenges through incident planning and risk mitigation.
15. Forde and Obrien (2022) - [60]	To address this gap and to provide recommendations for overcoming identified challenges.	Literature Review	29 articles	This literature review demonstrates the acceptability and usability of digitally enhanced practical teaching in health science education among students and educators.

## Data Availability

Not applicable.
